# The blind spot in high-dose tigecycline pharmacokinetics in critically ill patients: membrane adsorption during continuous extracorporeal treatment

**DOI:** 10.1186/s13054-015-0744-9

**Published:** 2015-01-28

**Authors:** Patrick M Honore, Rita Jacobs, Elisabeth De Waele, Viola Van Gorp, Herbert D Spapen

**Affiliations:** ICU Department, Universitair Ziekenhuis Brussel–Vrije Universiteit Brussel University, 101 Laarbeeklaan, Jette, Brussels 1090 Belgium

**Keywords:** ■■■

We read with interest the paper by De Pascale and colleagues [1] and the accompanying editorial [2] on high-dose tigecycline (TGC) in critically ill patients with severe infections due to multidrug-resistant bacteria. We agree that the currently recommended TGC dose (100 mg loading followed by 50 mg twice daily) may be largely insufficient for treatment of such infections and may promote resistance. In line with pharmacological logic, De Pascale and colleagues showed that increasing the TGC dose improved clinical outcome without enhancing toxicity. Interestingly, 25% of the patients included in their study received continuous renal replacement therapy. Details of and the distribution of continuous renal replacement therapy between groups were not provided.

TGC is a small lipophilic antibiotic with a high distribution volume in critically ill patients [3,4]. The relatively high protein binding of TGC precludes significant removal by intermittent hemodialysis [4]. Recent investigations suggest significant adsorption of many antimicrobial drugs on dialysis or extracorporeal membrane oxygenation membranes [3]. Moreover, the increasing use of highly adsorptive membranes for continuous hemo(dia)filtration may dramatically alter the pharmacokinetic behavior of many antibiotics [5,6]. Since this is particularly true for TGC, higher dose regimens (150 mg loading followed by 100 mg twice daily) in patients undergoing continuous renal replacement therapy and extracorporeal membrane oxygenation have been proposed [6].

De Pascale and colleagues are right to state that more pharmacokinetic investigation is needed before high-dose TGC can be recommended for treatment of multidrug-resistant bacterial infections [1]. We strongly suggest pursuing such research also in the growing cohort of critically ill patients treated with extracorporeal techniques equipped with sophisticated highly drug-adsorptive membranes.

## Abbreviation

TGC: Tigecyclin
